# Periocular Pigmentation After Gemcitabine Monotherapy for Bladder Cancer: A Rare Case Report

**DOI:** 10.1002/ccr3.72641

**Published:** 2026-05-13

**Authors:** Li Juan, Chen Weiping

**Affiliations:** ^1^ Macau University of Science and Technology Macau China; ^2^ The First Affiliated Hospital of Guizhou University of Traditional Chinese Medicine Guiyang China

**Keywords:** bladder cancer, drug adverse reaction, gemcitabine monotherapy, periocular pigmentation

## Abstract

A patient with advanced bladder cancer received antitumor therapy consisting of gemcitabine on days 1 and 8 combined with cisplatin and treliprimab on day 1. Severe periorbital hyperpigmentation occurred 30 min after gemcitabine infusion on day 8. Symmetrical diffuse hyperpigmentation of a dark brown hue with indistinct borders appeared around the affected eye, extending to the orbital and facial regions along the orbital margin. No accompanying symptoms, including pruritus, pain, or desquamation, were observed. The patient had no prior history of dermatological conditions and no family history of skin disorders. During chemotherapy, no other drugs, cosmetics, or specific foods known to induce skin hyperpigmentation were used. No additional abnormalities were identified during dermatological examination. Laboratory tests, including liver function indicators such as alanine aminotransferase (ALT) and aspartate aminotransferase (AST) within the range of 10–40 IU/L, kidney function parameters such as blood urea nitrogen (BUN) 2.8–7.1 mmol/L and creatinine 50–96 μmol/L, blood glucose levels of 3.9–6.1 mmol/L, thyroid function within normal limits, and electrolytes including sodium (Na) 135–145 mmol/L and potassium (K) 3.5–5.5 mmol/L, were all within their respective normal ranges. Considering the patient's medical background, treatment regimen, and the temporal relationship between the onset of symptoms and chemotherapy cycles, this periorbital hyperpigmentation likely represents a transient adverse reaction to gemcitabine, as supported by previously reported cases. Within 2 days after chemotherapy cessation, the periorbital hyperpigmentation improved significantly. This case highlights that during gemcitabine administration, clinicians should remain vigilant for rare adverse reaction of gemcitabine‐induced periorbital hyperpigmentation. Close skin monitoring throughout treatment is essential, and prompt evaluation and intervention should be carried out to improve the patient's quality of life.

## Key Clinical Message

Gemcitabine may rarely induce periorbital hyperpigmentation, which can be easily misdiagnosed as a primary skin disorder. Clinicians should closely monitor periorbital skin changes during chemotherapy. After excluding other etiologies, drug‐related adverse reactions should be considered, with timely follow‐up and appropriate intervention to avoid overtreatment.

## 1 Introduction

Gemcitabine, as one of the commonly used chemotherapeutic agents, is frequently employed in the treatment of non‐small cell lung cancer, pancreatic cancer, breast cancer, bladder cancer, and other malignancies. It, when combined with cisplatin and immune checkpoint inhibitors, has shown synergistic effects in the treatment of locally advanced or metastatic urothelial carcinoma, including bladder cancer, potentially enhancing the immune system's ability to recognize and attack cancer cells.

The common adverse reactions of gemcitabine are as follows: common adverse reactions (incidence > 30%) include myelosuppression, manifested as neutropenia, thrombocytopenia, and anemia; nausea, vomiting, and anorexia; fatigue and exhaustion; peripheral edema; elevated transaminases; rash and alopecia; and non‐infectious fever. Severe adverse reactions include myelosuppression, which may result in severe neutropenia and thrombocytopenia, thereby increasing the risk of infection and bleeding; pulmonary toxicity, presenting as interstitial pneumonia, alveolar hemorrhage, and acute respiratory distress syndrome (ARDS), with occasional fatal cases; hemolytic uremic syndrome (HUS), manifesting as microangiopathic hemolytic anemia, thrombocytopenia, and acute renal failure, requiring immediate discontinuation and active management; hepatotoxicity, characterized by significant elevation of transaminases, with rare cases of liver failure; nephrotoxicity, reflected by elevated creatinine levels, particularly when combined with cisplatin; allergic reactions, including chills, fever, bronchospasm, and hypotension, mostly occurring during the first or second administration; cardiovascular effects, such as arrhythmias, myocardial ischemia, and heart failure (rare but severe); and flu‐like syndrome, including fever, chills, headache, and myalgia, which are typically transient and self‐limiting. However, there are currently no reports describing adverse reactions related to periorbital hyperpigmentation, with previous studies mainly documenting rash manifestations.

The antitumor mechanism of gemcitabine is as follows: after entering the cell, it is phosphorylated by deoxycytidine kinase (dCK) to generate active metabolites, namely dFdCDP and dFdCTP. dFdCDP inhibits ribonucleotide reductase (RNR), thereby reducing the pool of deoxynucleotides and impairing DNA synthesis. dFdCTP is incorporated into the DNA strand, triggering “masked chain termination,” which allows the addition of a normal nucleotide prior to termination and thereby evades repair mechanisms. These two mechanisms result in DNA replication arrest and induce apoptosis, primarily acting on the S‐phase cells, and they exhibit cell cycle specificity.

## 2 Case History

**FIGURE 1:**
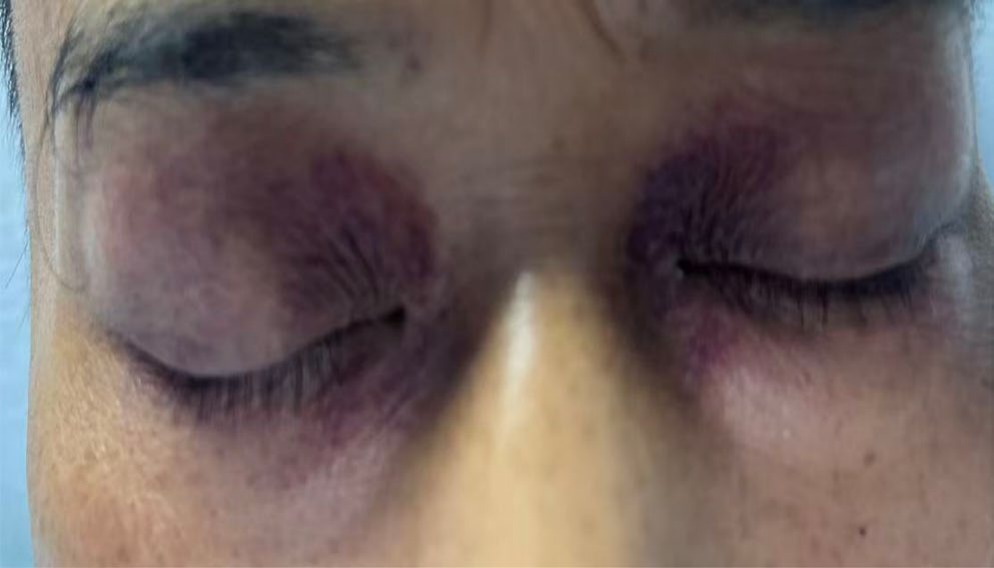
Severe periorbital hyperpigmentation occurred half an hour after gemcitabine monotherapy on day 8 of chemotherapy.

The patient, a middle‐aged male with a history of bladder urothelial carcinoma for over a year, was admitted for his initial chemotherapy cycle. In October 2024, the patient presented with urinary frequency, urgency, and dysuria without obvious precipitating factors, and did not experience nausea, vomiting, chills, fever, palpitations, chest tightness, abdominal pain, or diarrhea. He was subsequently referred to the hospital. Enhanced CT of the urinary system revealed a bladder mass suggestive of bladder cancer, with multiple pelvic lymph nodes showing increased density. Cystoscopy and pathological examination confirmed the presence of a bladder neoplasm, specifically a high‐grade urothelial carcinoma, with urethral invasion on the left side of the bladder wall. (Pathological code: G2409425): The patient was diagnosed with high‐grade papillary urothelial carcinoma of the urethra, characterized by fragmented tissue samples. Immunohistochemical results were as follows: CKpan (+), CK (H) (+), CK5/6 (−), P40 (+), P63 (+), CK7 (+), CK20 (partially +), P16 (diffuse strong +), Her‐2 (3+), GATA‐3 (+), Desmin (+, indicating smooth muscle), CD34 (+, indicating blood vessels), P53 (diffuse +, mutant type), Ki‐67 (+, about 80%). The diagnosis was bladder urothelial carcinoma with metastasis to the periplastic lymph nodes, classified as stage T3bN1M0 IIIa. The patient was otherwise in good health, with no history of chronic diseases, drug allergies, or familial genetic disorders. After admission, relevant examinations were completed, including electrocardiogram (ECG), complete blood count (CBC), liver and kidney function tests, coagulation function tests, and thyroid function tests, which showed no significant abnormalities. The patient underwent the first cycle of chemotherapy combined with immunotherapy in our department. The specific regimen was as follows: “Treliparib injection 210 mg ivgtt D1 Q2w” in combination with “Gemcitabine hydrochloride for injection 1.4g ivgtt d1, d8 + Cisplatin 100 mg ivgtt d1, q3w.” The course of antitumor treatment proceeded smoothly. At present, the patient is on day 8 of the first cycle of gemcitabine chemotherapy and presented for evaluation. Upon admission, physical examination indicated stable body temperature, pulse, respiration, and blood pressure, with no jaundice, rash, or petechiae observed on the skin or mucous membranes. On the eighth day after admission, the patient received gemcitabine monotherapy at 1.4 g. Half an hour after chemotherapy, symmetrical brown pigmented lesions with indistinct borders, smooth surfaces, and no pruritus, pain, desquamation, or exudation were observed on the eyelids and periorbital skin (including the medial canthus, lateral canthus, and lower eyelid), as shown in Figure 1. The symptoms induced by chemotherapy gradually worsened throughout the night, without accompanying discomfort such as dry eyes, irritation, or itching. The patient reported no recent excessive exposure to sunlight, no use of new skincare products, and no administration of specific medications.

Auxiliary examinations, including routine blood tests, liver and kidney function assessments, electrolyte levels, and coagulation function tests, indicated values within normal ranges for all parameters. Ophthalmological examination showed bilateral visual acuity of 1.0, normal intraocular pressure, no conjunctival congestion, transparent cornea, no lens opacity, and no abnormalities detected on fundus examination.

## 3 Diagnosis and Differential Diagnosis

Based on the patient's history of receiving gemcitabine chemotherapy, the appearance of periorbital pigmentation after chemotherapy without any other identifiable cause, and physical examination and auxiliary tests showing no evidence of hepatobiliary diseases, endocrine disorders (e.g., adrenal insufficiency), dermatological conditions (e.g., melasma, Riehl's melanosis), or local ocular lesions leading to periorbital pigmentation, the diagnosis was established as “gemcitabine chemotherapy‐associated periorbital pigmentation”.

## 4 Treatment and Follow‐Up

**FIGURE 2:**
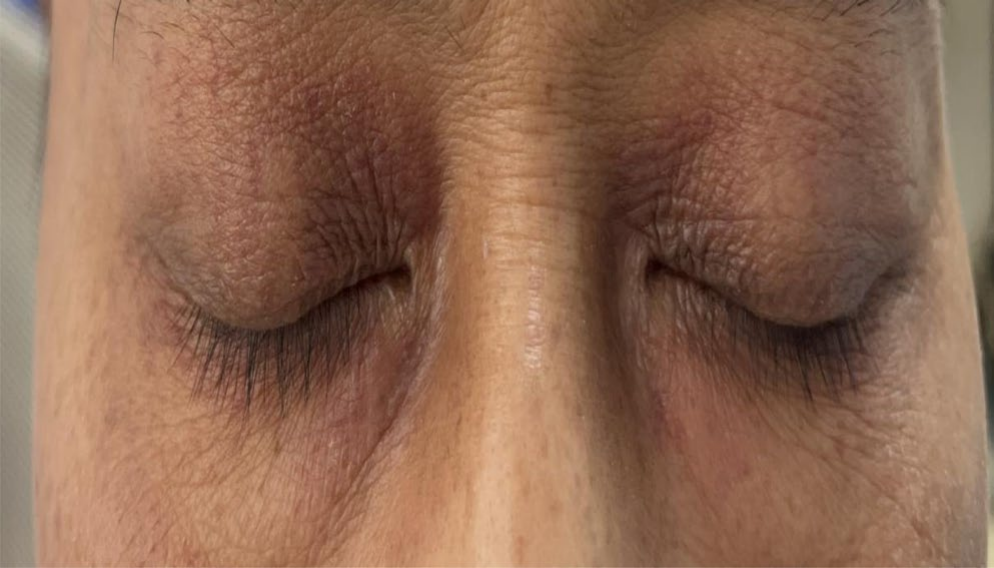
On the eighth day of first‐line antitumor therapy and the second day after gemcitabine monotherapy, periorbital pigmentation showed significant improvement compared with the previous day.

**FIGURE 3:**
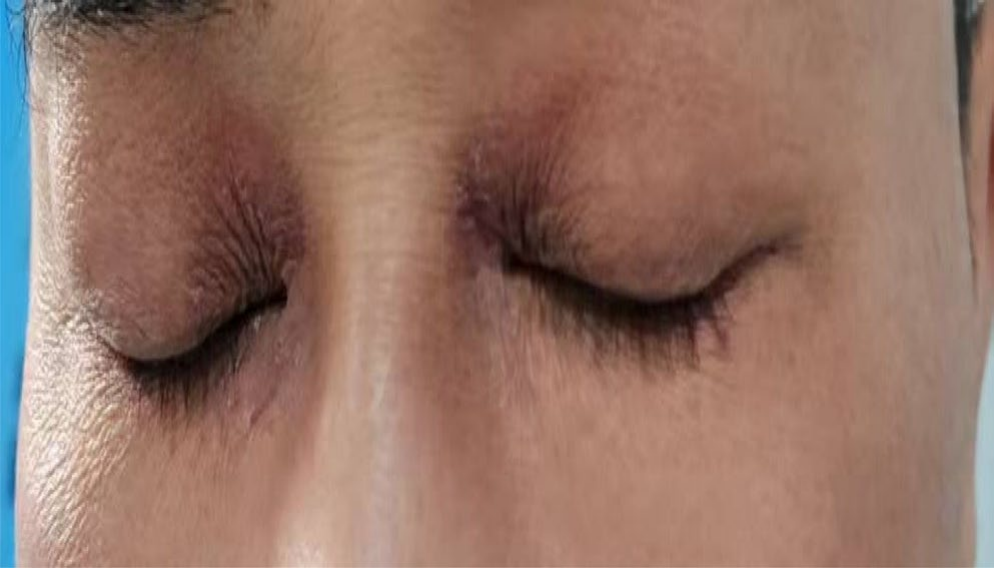
On the third day after chemotherapy, periorbital pigmentation approached normal levels.

Given that the current pigmentation represents only an aesthetic change without causing subjective discomfort or functional impairment, and gemcitabine treatment effectively controlled the patient's condition, no modifications to the chemotherapy regimen were made after completion of the first cycle. During the observation period, the patient was instructed to follow the subsequent health education recommendations: strengthen sun protection measures, avoid rubbing the periorbital skin, use mild and moisturizing skincare products, and seek prompt follow‐up if pigmentation worsens or ocular discomfort becomes more pronounced. Upon evaluation, the patient met the discharge criteria. On the second day following the first cycle of chemotherapy, the periorbital pigmentation gradually decreased (Figure 2), with no other abnormalities observed in the skin or ocular condition. By the third day after discharge, the periorbital pigmentation had further decreased (Figure 3). The patient was advised to undergo regular follow‐up.

## 5 Discussion

Gemcitabine, a pyrimidine‐based antimetabolite, is a key agent targeting DNA synthesis and is widely used in the treatment of various cancers. Clinical studies have shown its efficacy in treating bladder cancer, with combination therapies such as gemcitabine plus cisplatin demonstrating response rates of 56.1% [1]. Furthermore, studies suggest that the mechanism of action involves inhibiting DNA synthesis and repair, leading to cell death, and it has been found to be effective in both primary and metastatic lesions, with better outcomes in treatment‐naive patients [1]. Common adverse effects of gemcitabine include bone marrow suppression, gastrointestinal symptoms, and fatigue. Cutaneous reactions are less common, primarily presenting as alopecia, rash, and pruritus. Pigmentation is seldom reported, usually confined to localized periorbital hyperpigmentation, with very few documented cases.

The precise mechanism through which gemcitabine induces cutaneous hyperpigmentation is still unclear. It is generally proposed that the drug and its metabolites accumulate in skin tissues, directly stimulating melanocyte proliferation or the enhancement of tyrosinase activity, thereby increasing melanin synthesis. Alternatively, the chemotherapeutic agent may damage basal skin cells and trigger inflammatory responses, whereby inflammatory mediators activate melanocytes. Another possible mechanism involves drug‐induced disturbance of local microcirculation leading to cutaneous hypoxia, which in turn indirectly contributes to melanin deposition. Additionally, the periorbital skin, being thinner and more densely populated with melanocytes, may be more susceptible to gemcitabine‐induced hyperpigmentation.

It should be noted that current reports on gemcitabine‐induced hyperpigmentation mainly describe systemic hyperpigmentation or localized pigmentation of the hands, feet, and nails [2, 3, 4, 5, 6, 7, 8]. Both domestic and international literature include only a limited number of reports describing localized periorbital hyperpigmentation. In this case, the periorbital hyperpigmentation showed symmetrical bilateral distribution, was temporally related to chemotherapy, and presented with distinct clinical features. Clinicians should increase awareness of this rare adverse reaction, inform patients about potential cutaneous effects before gemcitabine chemotherapy, and advise close monitoring during treatment to enable early identification and appropriate management. In most cases, such hyperpigmentation does not require discontinuation of chemotherapy and tends to resolve gradually after treatment, although individual responses may vary. If hyperpigmentation significantly affects the patient's daily life and work, topical hydroquinone cream or laser depigmentation therapy may be considered after chemotherapy, although further clinical evidence is required to confirm their efficacy and safety.

In conclusion, periorbital hyperpigmentation is a rare cutaneous adverse reaction associated with gemcitabine chemotherapy. Clinicians should remain vigilant regarding the potential adverse effects of gemcitabine, closely monitor patients during treatment, and perform regular blood tests to avoid unnecessary examinations and interventions. In addition, patients should be adequately informed to alleviate concerns.

The patient's personal and relevant privacy information was concealed, and informed consent was obtained.

## Author Contributions

**Chen Weiping:** writing – review and editing. **Li Juan:** writing – original draft.

## Funding

This work was supported by the Macao Key R&D Funding Program, Project Number: 0001/2024/AKP2 and Macao University of Science and Technology Research Fund, Project Number: FRG‐24‐046‐FC. The funding bodies had no role in the design of the study and collection, analysis, and interpretation of data and in writing the manuscript.

## Ethics Statement

Ethical approval for this study was obtained from the institutional ethics committee or relevant ethics review board in accordance with the principles of the Declaration of Helsinki.

## Consent

Written informed consent was obtained from the patient.

## Conflicts of Interest

The authors declare no conflicts of interest.

## Data Availability

This study is a case report and does not include shareable raw datasets. All relevant data can be accessed in the original article.
